# Exploring the causal role of plasma metabolites and metabolite ratios in prostate cancer: a two-sample Mendelian randomization study

**DOI:** 10.3389/fmolb.2024.1406055

**Published:** 2025-01-06

**Authors:** Changzhou Feng, Haining Li, Chu Zhang, Ying Zhou, Huanhuan Zhang, Ping Zheng, Shaolin Zhao, Lei Wang, Jin Yang

**Affiliations:** Department of Clinical Laboratory, The First People’s Hospital of Lianyungang, The Affiliated Lianyungang Hospital of Xuzhou Medical University, The First Affiliated Hospital of Kangda College of Nanjing Medical University, Lianyungang, Jiangsu, China

**Keywords:** metabolites, metabolites ratios, prostate cancer (PCa), risk, Mendelian randomization (MR)

## Abstract

**Background:**

Prostate cancer (PCa), the most prevalent malignant neoplasm in males, involves complex biological mechanisms and risk factors, many of which remain unidentified. By employing a novel two-sample Mendelian randomization (MR) approach, this study aims to elucidate the causal relationships between the circulating metabolome and PCa risk, utilizing comprehensive data on genetically determined plasma metabolites and metabolite ratios.

**Methods:**

For the MR analysis, we utilized data from the GWAS Catalog database to analyze 1,091 plasma metabolites and 309 ratios in relation to PCa outcomes within two independent GWAS datasets. The inverse variance weighted (IVW) method was the primary approach for determining the existence of the causal relationship, supplemented by additional MR methods for heterogeneity, pleiotropy, and cross-validation. The false discovery rate (FDR) and Bonferroni correction were applied to identify the most significant causative associations. Additionally, reverse MR and Steiger filtering were conducted to ascertain whether PCa influenced the observed metabolite levels. Furthermore, metabolic pathway analysis was conducted with MetaboAnalyst 6.0 software.

**Results:**

In the MR analysis, our findings reveal three overlapped metabolite ratios (arginine to glutamate, phosphate to uridine, and glycerol to mannitol/sorbitol) inversely associated with PCa risk. Following FDR correction (FDR < 0.05), cysteinylglycine disulfide was identified as a potential reducer of PCa risk, whereas Uridine and N-acetyl-L-glutamine (NAG) were pinpointed as potential risk factors. Notably, NAG (OR 1.044; 95% CI 1.025–1.063) emerged as a metabolite with significant causal influence, as confirmed by stringent Bonferroni correction (*P* < 0.05/1400). Steiger’s directionality test (*P* < 0.001) and reverse MR confirmed the proposed causal direction. Furthermore, metabolic pathway analysis revealed a significant association between the “Glutathione Metabolism” pathway and PCa development.

**Conclusion:**

This study provides novel insights into the potential causal effects of plasma metabolites and metabolite ratios on PCa. The identified metabolites and ratios could serve as candidate biomarkers, contributing to the elucidation of PCa’s biological mechanisms.

## 1 Introduction

Prostate cancer (PCa) is projected to be the most frequently diagnosed cancer (29%) and the second leading cause of cancer-related mortality (11%) among males in the United States in 2024 (Siegel et al., 2024), as well as a significant cause of mortality worldwide (Sung et al., 2021). The effective prevention and treatment of PCa are crucial in reducing morbidity and mortality. Recent epidemiological studies have identified multiple genetic, lifestyle, and environmental factors associated with PCa risk (Maomao et al., 2022; Jiang et al., 2023). Notably, an increasing number of genetic susceptibility variants involved in the biological mechanisms of PCa have been identified through Genome-Wide Association Studies (GWAS) (Chen et al., 2024). However, these factors alone cannot fully explain the etiology and biological mechanisms of PCa. Moreover, the complex interplay among these factors complicates the determination of potential causality underlying their associations with PCa risk.

Circulating plasma metabolites, small molecules originating from cells, tissues, and biological fluids, encompass a variety of compounds such as amino acids, peptides, carbohydrates, lipids, and xenobiotics. It is noteworthy that metabolomics can offer novel insights into the biological mechanisms of diseases by revealing intermediate metabolites and altered metabolic pathways, a technique frequently employed to study physiological and pathophysiological processes in cancer (Chen et al., 2024; Wishart, 2019; Irajizad et al., 2023; Pavlova et al., 2022; Pan et al., 2024). Several studies have suggested that metabolites, as functional intermediates, can elucidate potential biological mechanisms related to the genetics of PCa (Chen et al., 2024; Lin et al., 2022). However, previous studies have predominantly focused on a limited subset of metabolites and were constrained by small sample sizes, potential confounders, and reverse causality. Moreover, many metabolites, including enzymes, transporters, substrates, and products of enzymatic reactions, reflect biological processes that individual plasma metabolites alone cannot reveal. Investigating cancer risks through metabolite ratios, such as substrate-to-product or metabolite pairs sharing an enzyme or transporter, warrants consideration (Chen et al., 2023). A recent mediation Mendelian randomization (MR) study indicated that the succinate-to-acetoacetate ratio mediated the effect of CD62L-monocyte % monocyte on PCa, with a mediation proportion of 16.6% (95% CI: −163%–196%) (Wu et al., 2024).

MR analysis, an epidemiological research strategy, utilizes genetic variants as instrumental variables (IVs) to link exposure with outcome, thereby assessing causal relationships. Compared to other epidemiological research strategies, MR offers unbiased estimates on genotypes determined at conception, which are not commonly susceptible to confounding factors and reverse causality (Hemani et al., 2018). Owing to this significant advantage, MR has been extensively applied over the past decade to infer the causality of risk exposures to diseases, utilizing publicly available GWAS summary statistics (Lin et al., 2024; Dai et al., 2024; Li et al., 2022). Recently, Chen YH et a (Chen et al., 2023) extended the comprehensive genomic atlas of the plasma metabolome, prioritizing metabolites implicated in human diseases, including 1,091 metabolite levels and 309 metabolite ratios. Additionally, Wang A et al. (Wang et al., 2023) reported the most recent GWAS summary statistics on PCa, featuring the largest sample size to date (726,828).

Herein, we hypothesize that this genetically determined 1,400 plasma metabolome atlas could elucidate the causality of the plasma metabolome on PCa. Consequently, we implemented a two-sample MR approach to assess the causal effects of the human plasma metabolome on PCa and identify potential metabolic pathways, which might elucidate the mechanism of PCa. To validate the significant causal associations identified, we conducted a series of complementary analyses to reinforce their reliability and robustness.

## 2 Materials and methods

### 2.1 Study design

**FIGURE 1:**
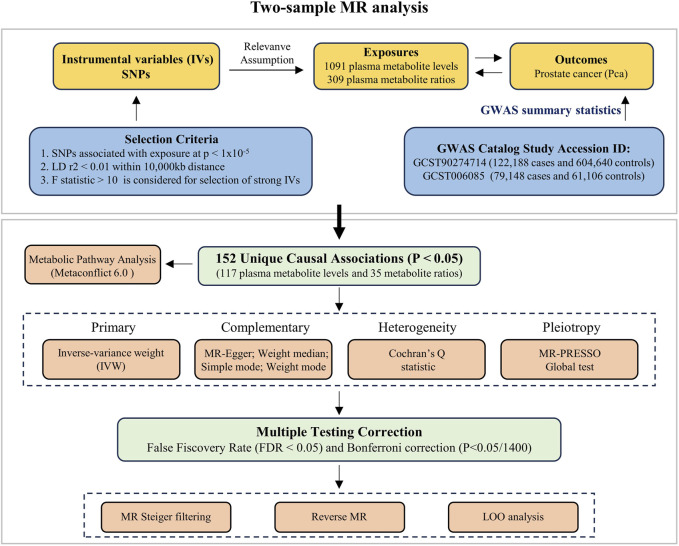
The overall study workflow of MR analysis.

This study systematically investigates the causal relationship between the plasma metabolome (exposures) and PCa (outcome) using MR. The methods of this study were in compliance with the STROBE-MR checklist (Skrivankova et al., 2021). Three foundational assumptions necessary for MR analysis were satisfied: (1) genetically determined IVs are strongly associated with the plasma metabolome; (2) the genetic IVs are not associated with any confounding factors related to the plasma metabolome and PCa; and (3) the genetic IVs influence PCa only through their effect on the plasma metabolome. This study utilized publicly available datasets which had already received ethical approval and informed consent; thus, no additional ethical declaration or consent was required. An overview of this study is depicted in Figure 1.

### 2.2 Data sources

To mitigate non-independence arising from the overlap of exposure and outcome samples, datasets for exposure and outcome were sourced from different databases. In selecting exposure and outcome GWAS summary statistics, criteria encompassed the European origin of the population, the larger size of population samples and SNPs, and the time of data publication. Recently, Chen et al. (Chen et al., 2023) reported a highly comprehensive exploration of the genetic influences on the plasma metabolome, with a focus on metabolites implicated in human diseases. In their study, a total of 8,299 unrelated individuals of European ancestries were recruited from the Canadian Longitudinal Study on Aging (CLSA) cohort (Raina et al., 2019), all of whom had genome-wide genotyping and measurements of circulating plasma metabolites. Following rigorous quality control and data curation, 1,091 metabolite levels were quantified in plasma samples by Metabolon, Inc. (https://www.metabolon.com/) using the Ultrahigh Performance Liquid Chromatography-Tandem Mass Spectroscopy (UPLC-MS/MS) platform. Of these, 850 known metabolites were categorized into eight super pathways: lipid, amino acid, nucleotide, xenobiotics, cofactor and vitamins, carbohydrate, peptide, and energy. Furthermore, to elucidate biological processes not apparent from the study of individual plasma metabolites alone, 309 genetic determinants of metabolite ratios were calculated, including substrate to product ratios and metabolite pairs sharing an enzyme or transporter. Notably, the most recent GWAS summary statistics for PCa, with the largest samples to date (122,188 cases; 604,640 controls; 25,146,978 SNPs) (Wang et al., 2023), was selected for the primary MR analysis. To validate the conclusions drawn, GWAS data were obtained from the Prostate Cancer Association Group to Investigate Cancer Associated Alterations in the Genome (PRACTICAL) Consortium, comprising 79,148 cases, 61,106 controls, and 19,716,640 SNPs (Schumacher et al., 2018). An overview of the GWAS summary statistics utilized in this study is available in the GWAS Catalog (https://www.ebi.ac.uk/gwas/) (Table 1).

**TABLE 1:** GWAS summary statistics used in this study.

GWAS Catalog (study accession ID)	Phenotype	Sample size (nCase/nControl)	nSNPs	Year	Population
GCST90274714	Prostate cancer (PCa)	726,828 (122,188/604,640)	25,146,978	2023	European
GCST006085		140,254 (79,148/61,106)	19,716,640	2018	
GCST90199621 - GCST90200711	1,091 Metabolite levels			2023	
GCST90200712 - GCST90201020	309 Metabolite ratios				

### 2.3 Selection of instrumental variables (IVs)

To identify the IVs for metabolite levels and ratios, several procedures were undertaken to verify the validity of the first assumption. Initially, genetic variants were identified through association thresholds of *p* < 1 × 10^−5^, a standard practice in MR analysis to encompass a broader range of variation when SNPs available for exposure are limited (Liu et al., 2024). Subsequently, SNPs showing independent inheritance and minimal linkage disequilibrium (LD) (r^2^ < 0.001, kb = 10,000) within the 1,000 Genomes European subset (Genomes Project et al., 2015) were identified through a clumping procedure in R software. SNPs that were ambiguous or palindromic were also excluded. To mitigate the risk of weak instrument bias, the F statistic (F > 10) was employed to ascertain if the identified IVs possessed sufficient power to represent metabolites or metabolite ratios (Burgess et al., 2011). We also utilized the NHGRI-EBI GWAS Catalog (www.ebi.ac.uk/gwas), which adheres to the findable, accessible, interoperable, and reusable (FAIR) data principles, to determine whether selected SNPs (or those in LD with them) were previously associated with other traits or diseases (*P* < 1 × 10⁻⁵) (Sollis et al., 2023). Using the LDtrait tool, SNPs associated with potential confounders, such as type 2 diabetes mellitus, non-PCa cancer, smoking, alcohol use, stroke, autoimmune diseases, coronary artery disease, and hyperlipidemia, were excluded to minimize confounding between significantly associated metabolites and PCa. To augment the reliability of our findings, an online power calculator (mRnd) was utilized to calculate the statistical power for MR (https://cnsgenomics.shinyapps.io/mRnd/).

### 2.4 MR analyses

In this study, five complementary MR methods were utilized to estimate the causal relationship between exposure and outcome, namely MR-Egger (Bowden et al., 2015), weighted median (Bowden et al., 2016), inverse variance weighted (IVW), simple mode, and weighted mode. Among these, the IVW method, which provides the most accurate causal estimation between exposure and outcome, served as the primary approach (Burgess et al., 2013). Furthermore, several supplementary analyses were performed to validate the robustness of our MR results. The MR-Egger method not only identifies violations of the IVs assumption but also calculates intercepts to assess potential horizontal pleiotropy and bias from ineffective IVs, providing effect estimates unaffected by these violations (Bowden et al., 2015). Cochran’s Q statistic was applied to estimate heterogeneity among the variables (SNPs) (Cohen et al., 2015). Moreover, the MR-PRESSO global test, an additional MR method designed to identify and correct horizontal pleiotropic outliers, was utilized to assess the existence of horizontal pleiotropy (Verbanck et al., 2018). A “leave-one-out (LOO)” sensitivity analysis was conducted to determine whether results were affected by a single SNP (Burgess and Thompson, 2017). Finally, scatter plots and funnel plots were used to visually display the relationships and interplay between each genetic instrument.

Notably, all analyses utilized the “TwoSampleMR” (version 0.4.22) and MR-PRESSO packages in R software (version 3.6.0). A *P*-value of <0.05 was deemed statistically significant. Moreover, in light of multiple testing concerns, the false discovery rate (FDR) and Bonferroni correction were applied to identify statistically significant results in multiple comparisons. According to prior studies, FDR < 0.05 and Bonferroni correction are regarded as indicative of significant causal relationships, with Bonferroni correction being notably more stringent.

### 2.5 Reverse Mendelian randomization

To investigate whether the researched outcomes had an impact on the plasma metabolome, a reverse MR analysis was performed. In this reverse analysis, SNPs (*P* < 5 × 10^−8^, r^2^ < 0.001, kb = 10,000) selected from GWAS data on PCa as IVs, with blood plasma metabolite levels as the outcome, were used to assess the bidirectionality of the previously determined causal relationship.

### 2.6 Metabolic pathway analysis

Metabolic pathway analysis was conducted using the online tool Metaconflict 6.0 (https://www.metaboanalyst.ca/) (Chong et al., 2018). Functional enrichment analyses and the pathway analyses module were utilized to identify metabolite groups or pathways potentially relevant to the biological processes underlying PCa. Importantly, this study focused solely on metabolites that surpassed the recommended association threshold as determined by IVW (*P*_IVW-mre_ < 0.05).

### 2.7 Glutathione metabolism-related gene set enrichment analysis (GSEA)

Gene expression data were obtained from the publicly available microarray dataset GSE46602, hosted in the Gene Expression Omnibus (GEO) database (http://www.ncbi.nlm.nih.gov/geo). This dataset consists of 14 benign prostate gland samples and 36 PCa samples (Mortensen et al., 2015). Subsequently, Gene Set Enrichment Analysis (GSEA) was conducted using the R package “clusterProfiler” to identify significantly enriched biological pathways. Given the focus of our study, we specifically examined gene sets related to glutathione metabolism. The analysis was performed using default parameters, and the resulting *p*-values were adjusted using the FDR method to control for multiple hypothesis testing.

## 3 Results

### 3.1 Strength of the instrumental variables (IVs)

After a rigorous selection process, MR analysis was conducted to evaluate the causality of 1,091 metabolite levels and 309 metabolite ratios on PCa using independent GWAS summary datasets. The IVs generated for the plasma metabolome ranged from 11 to 93 SNPs, with a minimum F statistic of 19.50, indicating that all IVs were sufficiently robust for MR analysis. Supplementary Tables S1, S2 displays the characteristics of SNPs and their genetic associations with the 1,400 plasma metabolome and PCa.

### 3.2 Overview of MR analysis results among plasma metabolome on PCa

**FIGURE 2:**
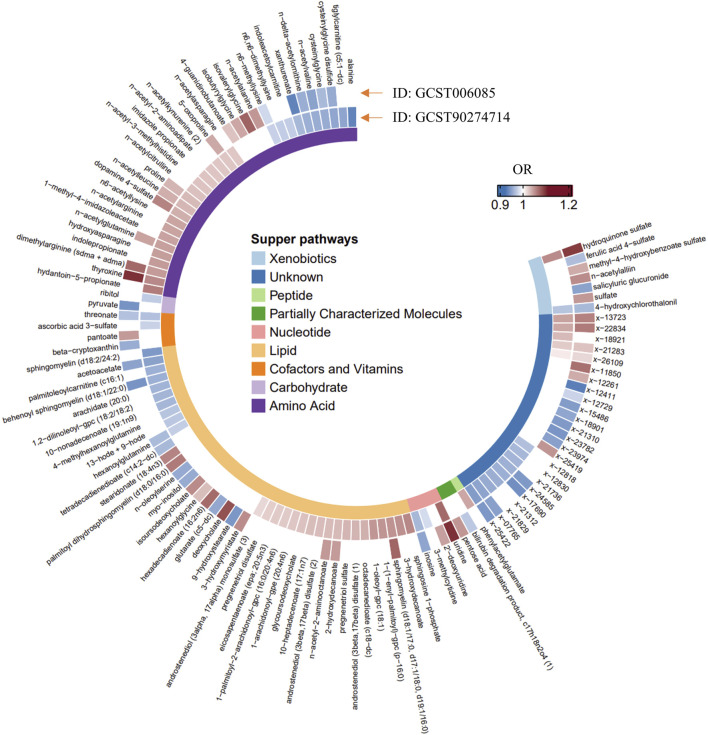
Mendelian randomization associations of 117 plasma metabolite levels on the risk of the two different GWAS datasets of PCa (derived from the random-effect IVW analysis). OR, Odds Ratio.

The IVW method of MR was utilized to establish causality between the 1,400 plasma metabolome components and PCa, using independent GWAS summary data. A total of 152 suggestive and unique causal associations (*P*_IVW-mre_ < 0.05) were identified, comprising 117 plasma metabolite levels and 35 metabolite ratios, as detailed in Supplementary Tables S3, S4. Of the 117 plasma metabolites, 91 were identified as known metabolites (including 40 lipids, 33 amino acids, 7 xenobiotics, 4 nucleotides, 4 cofactor and vitamins, 2 carbohydrates, and 1 peptide), while 26 were unknown or partially characterized, as classified by the Kyoto Encyclopedia of Genes and Genomes (KEGG) database (Kanehisa and Goto, 2000). Importantly, all overlapping components of the plasma metabolome exhibited consistent causal effects on PCa across various GWAS dataset pairs, as shown in Figure 2. Additionally, the outcomes of heterogeneity analysis employing Cochran’s Q statistic (applied to both IVW and MR-Egger methods) and pleiotropy analysis using MR-Egger intercepts and the MR-PRESSO global test for these suggestive causal associations are detailed in Supplementary Tables S5, S6.

### 3.3 Causality of genetically determined metabolite ratios on PCa

**FIGURE 3:**
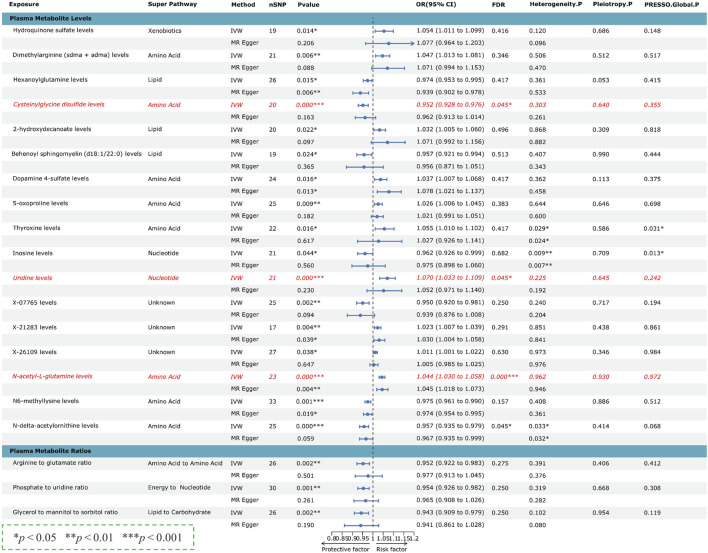
Forest plots presenting the causal effects of 20 identified overlapping plasma metabolites and metabolite ratios on prostate cancer (PCa) risk. Three metabolites, highlighted in red fonts, surpassed the FDR correction threshold (FDR < 0.05), exhibiting no pleiotropy or heterogeneity. SNPs, Single nucleotide polymorphisms; OR, odds ratio; CI, confidence interval; FDR, *P*-value corrected by FDR.

To confirm the robustness of our findings, further screening was performed on the 152 suggestive associations. Associations within the plasma metabolome consistently identified across all five complementary MR methods (IVW, simple mode, weighted mode, weighted median, and MR-Egger) were included. Figure 3 demonstrates that 20 causal associations were consistent across two distinct GWAS datasets of PCa. All three causative associations among the overlapped metabolite ratios were negatively correlated with PCa risk, specifically: arginine to glutamate ratio, phosphate to uridine ratio, and glycerol to mannitol/sorbitol ratio, with corresponding odds ratios (OR) and confidence intervals detailed in the text. Additionally, no evidence of pleiotropy or heterogeneity was observed among these associations. However, after applying FDR correction to these *P*-values, no significant confirmative associations were observed, categorizing the results as suggestive causal associations (Figure 3).

### 3.4 Causality of genetically determined metabolite levels on PCa

**FIGURE 4:**
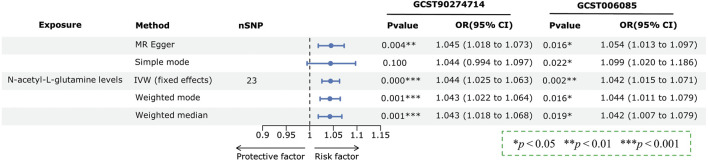
Forest plots depicting the causal effect estimates of N-acetyl-L-glutamine (NAG) levels on prostate cancer (PCa) risk across two independent datasets, utilizing five complementary MR methods.

Among the 17 overlapping metabolite levels, 14 causative associations passed all sensitivity analyses without evidence of heterogeneity or horizontal pleiotropy, with three showing significant association with PCa risk (FDR < 0.05). Significant associations were observed with cysteinylglycine disulfide levels negatively correlated with PCa risk, and uridine and N-acetyl-L-glutamine (NAG) levels positively correlated, as detailed with corresponding OR and confidence intervals in Figure 3. Bonferroni correction (*P* < 0.05/1,400; 3.571 × 10^−5^) was utilized to identify the most significant causal relationships among the 1,400 plasma metabolome components and PCa. Results indicated that NAG levels are significantly associated with an increased risk of PCa. The statistical power for the association of NAG levels with PCa was 1.0, demonstrating robustness against a type I error rate of 0.05. Fixed-effect IVW estimates confirmed the association of NAG levels with an increased risk of PCa across two datasets. Specifically, additional methods including the weighted median, weighted mode, simple mode, and MR-Egger, indicated consistent results, as shown in Figure 4 and Supplementary Figure S1, S2. Furthermore, based on the “leave-one-out” analysis, none of the associations were driven solely by a single SNP, as evidenced in Supplementary Figures S3–S6. The MR Steiger directionality test results confirmed the accuracy of our causal direction estimate (*P* < 0.001). Reverse MR analysis showed no causal relationship from PCa to NAG levels, indicating directionality in the observed association, as detailed in Supplementary Table S7. Consequently, it was determined that NAG levels might be causally associated with PCa, underscoring the reliability of the results.

### 3.5 Metabolic pathway analyses

Metabolic pathway analysis identified five pathways significantly associated with the development of PCa (*P* < 0.05): Glutathione metabolism, Biosynthesis of unsaturated fatty acids, Arginine and proline metabolism, Tyrosine metabolism, and Pyrimidine metabolism. Notably, the Glutathione metabolism pathway emerged as a recurrent feature across two independent PCa datasets, underscoring its potential role in PCa pathophysiology (Table 2). To further delineate the molecular underpinnings of this finding, we performed GSEA focused on glutathione-related gene sets. This approach enabled a more integrated assessment of dysregulated metabolic signatures in PCa, transcending the metabolite-level observations. The GSEA results revealed a consistent downregulation of multiple glutathione metabolism-associated gene sets in malignant prostate tissues compared to benign controls. Among these, GOMF_Glutathione_Transferase_Activity and WP_Glutathione_Metabolism showed statistically significant enrichment patterns (FDR q < 0.05) (Supplementary Figure S7).

**TABLE 2:** Significant metabolic pathways involved in different GWAS datasets of PCa.

GWAS Catalog (study accession ID)	Metabolic pathway	Total	Expected	Hits	*P*-value
GCST90274714	Glutathione metabolism^a^	28	0.24889	2	<0.05
	Biosynthesis of unsaturated fatty acids	36	0.32	2	<0.05
GCST006085	Arginine and proline metabolism	36	0.32	3	<0.01
	Tyrosine metabolism	42	0.37333	3	<0.01
	Glutathione metabolism^a^	28	0.24889	2	<0.05
	Pyrimidine metabolism	39	0.34667	2	<0.05

^a^ The significant metabolic pathway overlapped.

## 4 Discussion

In this comprehensive MR analysis, leveraging large-scale GWAS data, we identified 152 significant associations. These suggest potential causal influences of the plasma metabolome—including 117 metabolite levels and 35 metabolite ratios—on the risk of PCa. Of these, 17 metabolite levels and three metabolite ratios exhibited consistent associations across two independent PCa datasets. Notably, following stringent Bonferroni correction, plasma NAG emerged as the most robust causal factor. Furthermore, integrating metabolite-focused MR findings with GSEA of transcriptomic data implicated glutathione-related metabolic pathways in PCa pathogenesis. Collectively, these results underscore the complex interplay between genetic variation, metabolic profiles, and PCa, and may inform the development of novel prevention and therapeutic strategies.

Effective methods for cancer risk assessment and prevention remain a paramount priority. Advances in metabolomics have heightened interest in circulating metabolites as non-invasive biomarkers that reflect both endogenous metabolic states and environmental exposures (Qiu et al., 2023). Certain metabolites have been implicated in tumor biology; for example, proline—a highly abundant amino acid in collagen—contributes to the formation of extracellular matrices by cancer-associated fibroblasts, thereby fostering a tumor-promoting microenvironment (Kay et al., 2022). Moreover, metabolites involved in nucleotide metabolism have shown potential as therapeutic targets, as their modulation can impede tumor progression in preclinical models (Stine et al., 2022). In the present study, the observed positive association between asymmetric dimethylarginine (ADMA), an endogenous nitric oxide synthase (NOS) inhibitor, and PCa risk is consistent with emerging molecular evidence (Reddy et al., 2018; Asha Parveen et al., 2023). Similarly, dopamine 4-sulfate, a dopamine metabolite implicated in tumor proliferation, was positively associated with PCa risk. Prior studies have indicated its potential as a predictive biomarker for non-muscle invasive bladder cancer (NMIBC) (Cheng et al., 2018), suggesting that it may exert broader oncogenic influences.

Among the 1,400 plasma metabolites and ratios analyzed, plasma NAG demonstrated the most pronounced causal relationship with PCa. Mechanistically, NAG is intimately linked to glutamine metabolism. Glutamine—a central amino acid supporting biosynthesis, bioenergetics, and cell signaling—furnishes cancer cells with essential nitrogen and carbon for the synthesis of nucleotides, amino acids, and lipids, while also maintaining redox balance (Pavlova et al., 2022). By undergoing N-acetylation, glutamine’s stability, transport, and downstream metabolic availability may be enhanced, potentially providing tumor cells with more refined control over glutamine-derived intermediates. This regulatory mechanism could bolster cancer cell growth, survival, and invasive capabilities (Caldovic et al., 2010). Moreover, within the tumor microenvironment, PCa cells frequently display metabolic reprogramming driven by oncogenic signals and genetic alterations, which optimize glutamine uptake and utilization. Studies in irradiation-resistant HepG2 cells have further highlighted elevated NAG levels under ferroptotic stress, suggesting a role for this metabolite in stress response pathways (Yuan et al., 2022). Although our MR analysis strongly implicates NAG in PCa etiology, additional experimental studies are warranted to elucidate the precise molecular mechanisms involved.

Consideration of multiple-testing adjustments strengthens the validity of our findings. Beyond the Bonferroni correction, FDR correction identified cysteinylglycine and uridine as significantly associated with PCa risk, acting as a protective and risk factor, respectively. Previous research has documented that elevated extracellular cysteinylglycine, a metabolite related to glutathione, can suppress ferroptosis in certain cancer contexts, thereby fostering tumor growth (Hayashima and Katoh, 2022). Elevated serum cysteinylglycine levels have also been associated with a heightened risk of breast cancer (Lin et al., 2007), although other studies have reported inverse or null associations with certain malignancies (Houghton et al., 2019; Miranti et al., 2016). Uridine, a pyrimidine nucleoside abundant in plasma, fulfills multiple biological roles, including those related to antioxidant capacity and aging (Lai et al., 2023; Zhang et al., 2022). Functionally, uridine may enhance cancer cell proliferation and survival by serving as an energy source and modifying protein O-GlcNAcylation patterns (Yang et al., 2024). Our MR results, which identify uridine as a risk factor for PCa, align with these mechanistic insights. Taken together, these findings suggest that NAG, cysteinylglycine, and uridine may influence PCa onset and progression, potentially through pathways involving oxidative stress or ferroptosis regulation.

To our knowledge, this study is the first MR investigation to integrate genomic data with plasma metabolite ratios to assess causality in PCa risk. Our analyses identified three metabolite ratios—arginine to glutamate, phosphate to uridine, and glycerol to mannitol/sorbitol—that were significantly and inversely associated with PCa risk across datasets. These ratios integrate multiple metabolic dimensions: for instance, arginine, as a precursor to nitric oxide, may enhance immune surveillance, whereas glutamate supports tumorigenic processes. A higher arginine-to-glutamate ratio could thus reflect metabolic conditions less conducive to cancer cell proliferation (Huang et al., 2019). Likewise, while phosphate contributes to ATP production and phosphorylation signaling, uridine supports RNA and nucleotide biosynthesis. Thus, a metabolic environment favoring phosphate over uridine may be less advantageous for the rapid proliferation of malignant cells (Pavlova et al., 2022; Lai et al., 2023). The glycerol-to-mannitol/sorbitol ratio may similarly indicate restricted use of alternative carbohydrate pathways by cancer cells, potentially limiting their ability to adapt to oxidative stress and altering their energy metabolism (Schwab et al., 2024).

Our pathway analyses indicate that alterations in glutathione metabolism may contribute to the redox imbalances, growth advantages, and oxidative stress resistance commonly observed in PCa. Glutathione, as a key intracellular antioxidant, is integral to maintaining cellular redox homeostasis. Tumors can exploit glutathione-related pathways to mitigate oxidative stress, enhance survival under challenging microenvironmental conditions, and promote therapeutic resistance. By modulating components of glutathione synthesis, recycling, or transport, it may be possible to disrupt this carefully orchestrated balance, rendering cancer cells more susceptible to oxidative damage. Agents designed to inhibit key enzymes, transporters, or regulatory factors within this pathway could diminish the cancer cells’ metabolic flexibility. When combined with standard treatments—such as androgen deprivation therapy, chemotherapy, or emerging targeted therapies—these metabolic interventions may lower the threshold for cancer cell death, delay resistance, and enhance long-term treatment efficacy.

This study is not without limitations. First, the accuracy of MR analyses is contingent upon the selection and interpretation of genetic instruments. The lack of sex-specific instruments and potential genetic heterogeneity may introduce residual confounding. Additionally, while MR offers robust evidence for causal inference, our findings require experimental validation to unravel the underlying biological mechanisms. These initial results, however, provide a foundation for future research. We advocate for enhanced screening protocols in populations with metabolic dysregulation and recommend longitudinal cohort studies to identify metabolite-based biomarkers of cancer recurrence. Such efforts may ultimately improve the clinical prevention, early detection, and prognosis of PCa.

## 5 Conclusion

This study reveals novel causal relationships between specific plasma metabolites and PCa risk, notably identifying N-acetyl-L-glutamine (NAG) as a key factor and underscoring glutathione metabolism as a pivotal pathway. By employing a robust two-sample MR framework, these findings illuminate the intricate metabolic underpinnings of PCa, thus offering valuable new insights into its pathophysiological mechanisms. Such revelations not only enrich our understanding of disease etiology but also point toward promising biomarkers for early detection, prevention, and targeted therapeutic strategies.

## Data Availability

The original contributions presented in the study are included in the article/Supplementary Material, further inquiries can be directed to the corresponding authors.
