# Supplementary material for: Exploring the causal role of plasma metabolites and metabolite ratios in prostate cancer: a two-sample Mendelian randomization study
Source: Front Mol Biosci. 2025 Jan 6;11:1406055. doi: 10.3389/fmolb.2024.1406055 (PMC11743260; doi:10.3389/fmolb.2024.1406055)
Supplement: Supplementary file 1 [file DataSheet2.pdf]

Supplementary Material

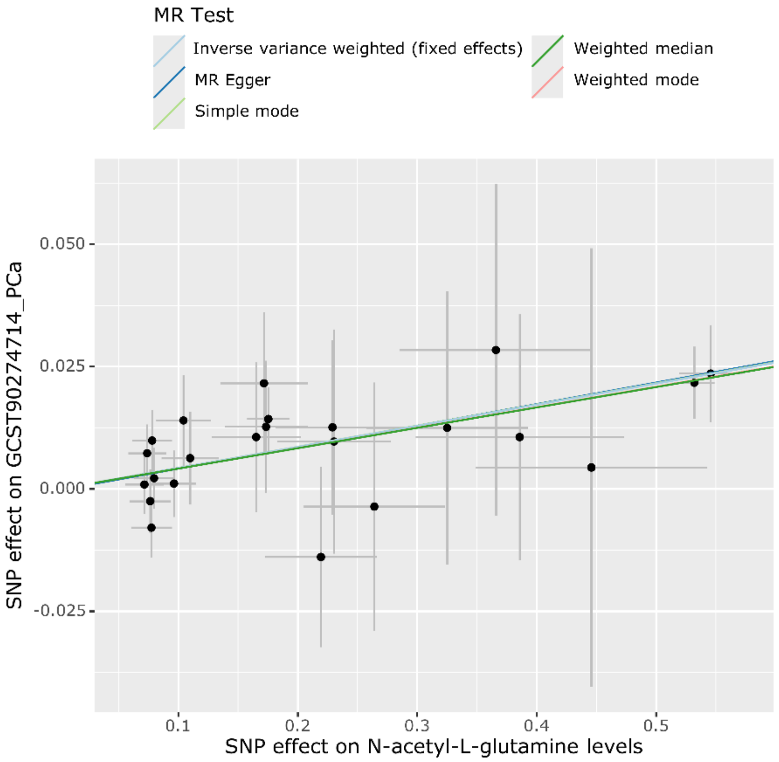

Supplementary Figure S1: Scatter plots of the causal association of N-acetyl-L-glutamine levels on the risk of Prostate cancer (PCa, GCST90274714).

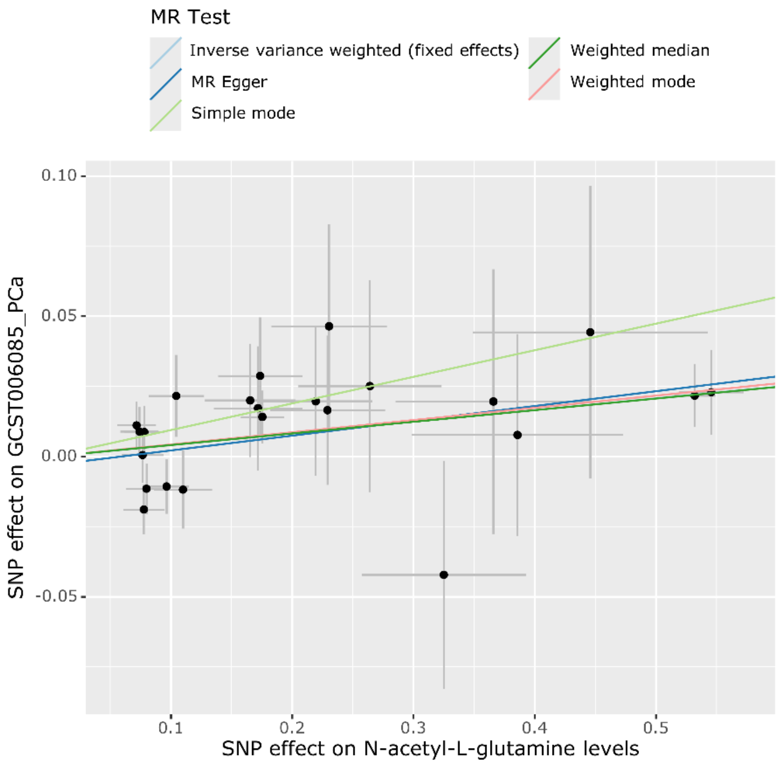

Supplementary Figure S2: Scatter plots of the causal association of N-acetyl-L-glutamine levels on the risk of prostate cancer (PCa, GCST006085).

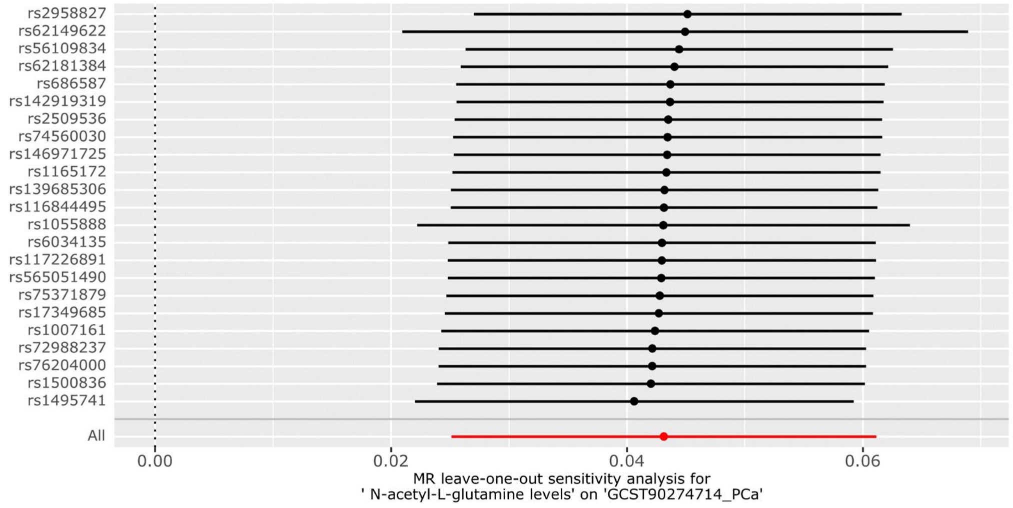

Supplementary Figure S3: Leave-one-out plots for N-acetyl-L-glutamine levels on prostate cancer (PCa, GCST90274714).

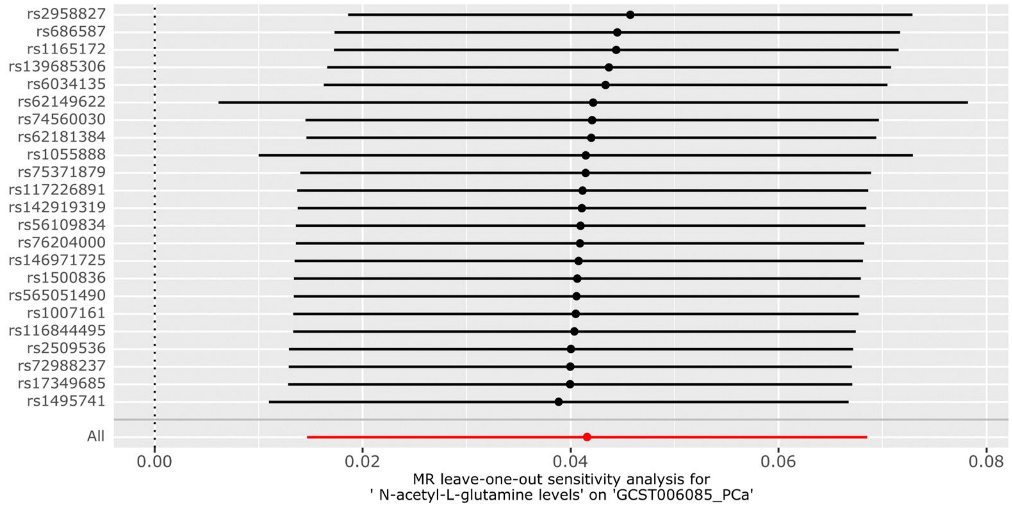

Supplementary Figure S4: Leave-one-out plots for N-acetyl-L-glutamine levels on prostate cancer (PCa, GCST006085).

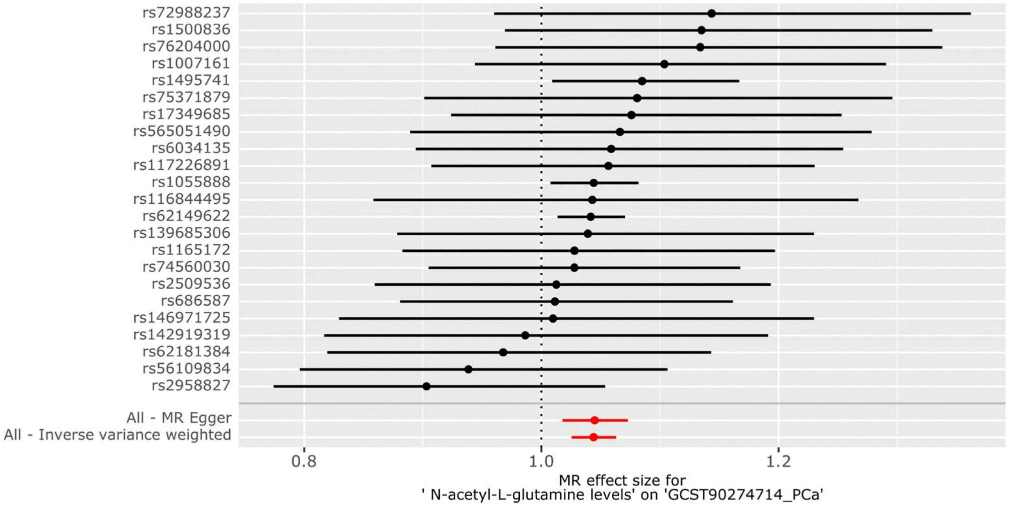

Supplementary Figure S5: Forest plots for N-acetyl-L-glutamine levels on prostate cancer (PCa, GCST90274714).

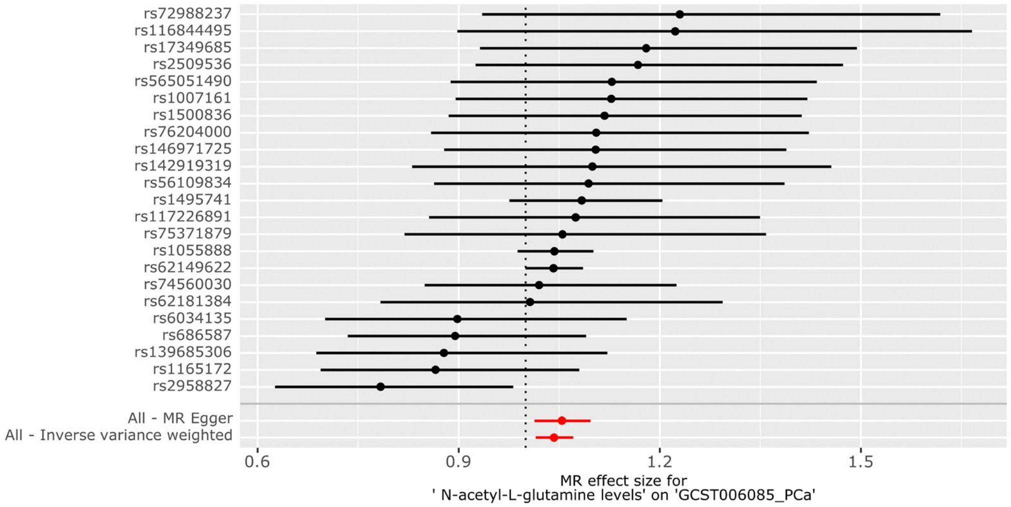

Supplementary Figure S6: Forest plots for N-acetyl-L-glutamine levels on prostate cancer (PCa, GCST006085).

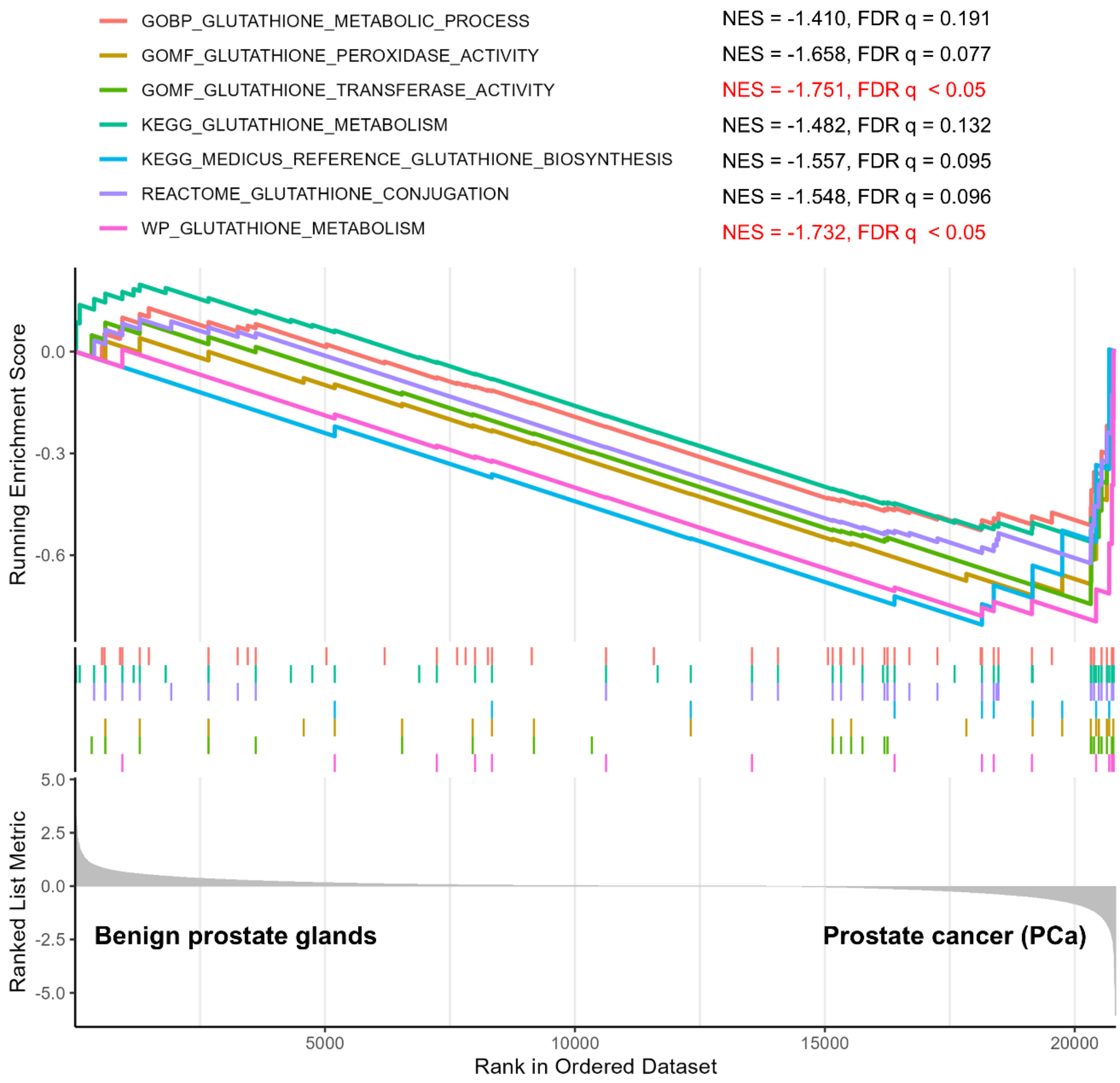

Supplementary Figure S7: Gene Set Enrichment Analysis (GSEA) using GSE46602 dataset. GSEA revealed significantly downregulated GOMF_Glutathione_Transferase _Activity and WP_Glutathione_Metabolism in PCa.
